# Early warning and diagnostic visualization of Sclerotinia infected tomato based on hyperspectral imaging

**DOI:** 10.1038/s41598-022-23326-2

**Published:** 2022-12-07

**Authors:** Yongxin Zhou, Jiaze Chen, Jinfang Ma, Xueqin Han, Bijuan Chen, Guilian Li, Zheng Xiong, Furong Huang

**Affiliations:** 1 Opto-Electronic Department of Jinan University, Guangzhou, 510632 China; 2 Guangdong Institute of Modern Agricultural Equipment, Guangzhou, 510630 China; 3 Guangdong Hongke Agricultural Machinery R&D Co., Ltd, Guangzhou, 510555 China

**Keywords:** Plant sciences, Optics and photonics

## Abstract

This research explored the feasibility of early warning and diagnostic visualization of Sclerotinia infected tomato by using hyperspectral imaging technology. Healthy tomato plants and tomato plants with *Sclerotinia sclerotiorum* were cultivated, and hyperspectral images at 400–1000 nm were collected from healthy and infected tomato leaves at 1, 3, 5, and 7 days of incubation. After preprocessing the spectra with first derivative (FD), second derivative (SD), standard normal variant (SNV), and multiplicative scatter correction (MSC) partial least squares discriminant analysis (PLS-DA) and support vector machine (SVM) were used to construct tomato sclerotinia identification model and select the best preprocessing method. On this basis, two band screening methods, competitive adaptive reweighted sampling (CARS) and successive projections algorithm (SPA), were introduced to reduce data redundancy and improve the model’s prediction accuracy. The results showed that the accuracy of the validation sets and operation speed of the CARS-PLS and CARS-SVM models were 87.88% and 1.8 s, and 87.95% and 1.78 s, respectively. The experiment was based on the SNV-CARS-SVM prediction model combined with image processing, spectral extraction, and visualization analysis methods to create diagnostic visualization software, which opens a new avenue to the implementation of online monitoring and early warning system for sclerotinia infected tomato.

## Introduction

Tomatoes are rich in antioxidant, ascorbic acid, and vitamin C^1^. In addition to being eaten alone, tomatoes can be processed into a variety of products, such as tomato ketchup and tomato powder, which are widely of consumed. Hence, tomatoes become one of the world's most popular agricultural products^2^. The growth process of tomatoes is susceptible to disturbance from external factors, such as nutrition, pests, and disease. Among them, disease is one of the biggest hazards during the process. *Sclerotinia sclerotiorum* (*S. sclerotiorum*), a fungal pathogen^3^, is one of the most common diseases affecting tomatoes. *S. sclerotiorum,* which can develop in plastic sheds, greenhouses, or outdoors, mainly affects stems, leaves, and fruits. In addition, *S. sclerotiorum* is a soil-borne fungal disease with a broad range of hosts, a high degree of genetic variability^4^, and can survive on the soil surface for 10 years^5^. Failure to detect and treat diseased tomatoes in a timely manner can have long-term and detrimental effects on large areas of tomato crops.

Health monitoring and timely disease detection are essential for effective morbidity control and tomato crop management^6^. Currently, the main means of monitoring tomato health involves the initial analysis of plant diseases through experience and then a combination of double-stranded RNA electrophoresis techniques^7^, polymerase chain reaction (PCR)^8^, flow cytometry (FCM)^9^, immunofluorescence (IF)^10^, enzyme-linked immunosorbent assay (ELISA)^11^, and other complex chemical laboratory analysis methods. On the one hand, these methods usually require experienced professionals to operate, which are inefficient and inconvenient to rapid diagnosis. On the other hand, these methods require expensive test equipment and consumption of chemical reagents, which pollute the environment and increase measurement costs.

Hyperspectral imaging combines imaging and spectroscopy by capturing one-dimensional spectral information (λ) and two-dimensional image information (x, y) in a three-dimensional data cube (x, y, λ). For each pixel point, there is a complete spectral curve, and for each band, there is a single wavelength figure. The images can cover both the visible light and near-infrared bands. Therefore, this method has been widely applied in non-destructive biological studies and crop studies concerning abiotic stresses in recent years^12,13^. For example, Huang et al.^14^ collected photochemical reflectance index by using airborne hyperspectral equipment to measure the accuracy of the disease index of yellow rust in wheat with the determination coefficient of up to 0.91. Xie et al.^15^ used hyperspectral imaging with spectral reflectance information and imaging features to classify early and late blight of tomato leaves. Xie et al.^16^ used hyperspectral imaging to classify healthy and infected tomato leaves according to the different infection levels of gray mold, where FN-KNN was the best model with the accuracy of 97.22%. Gu et al.^17^ achieved early detection of tomato spotted wilt virus via using hyperspectral imaging, in which the SPA-BRT algorithm was used for model construction with the accuracy of up to 93.2%. The above studies highlight the practicality and feasibility of using hyperspectral imaging technology for detecting crop diseases. However, there are few studies focused on tomato leaves infected with *S. sclerotiorum*. Due to the threat imposed by this disease, the use of hyperspectral technology to achieve the detection of *S. sclerotiorum* on tomato is of great significance for the effective tomato cultivation and disease prevention.

Since a hyperspectral image is a three-dimensional data cube, the collected data are a set of multi-band spectral data that often contain some degree of redundant information due to covariance between data. Therefore, many methods of wavelength selection, such as successive projections algorithm (SPA) and competitive adaptive reweighted sampling (CARS), can effectively improve the efficiency of data use and reduce the complexity of hyperspectral calculations^18^. On the other hand, hyperspectral imaging contains a large amount of two-dimensional image information, which facilitates spectral acquisition and prediction across large areas. For example, Xiao et al.^19^ used hyperspectral imaging to visualize the taxonomic results of Radix Astragali in five different regions, while Pu et al.^20^ visualized the moisture distribution results of different mango drying methods. The results of these studies could be presented in a more aesthetic and intuitive manner. This study developed hyperspectral imaging-based visualization software for early warning and diagnosis of sclerotinia infected tomato in preparation for online detection.

The objectives of this study were to: (1) investigate the feasibility of using hyperspectral imaging for the early detection of tomato *S. sclerotiorum* infection; (2) explore the effects of SPA and CARS band screening on the accuracy and efficiency of predictive models; (3) determine the best combination of band screening methods and predictive models for the early detection of sclerotinia infected tomato; (4) develop the early warning and diagnostic visualization software based on the hyperspectral imaging to detect the sclerotinia infected tomato.

## Results and discussion

### Spectral acquisition and properties

**Figure 1:**
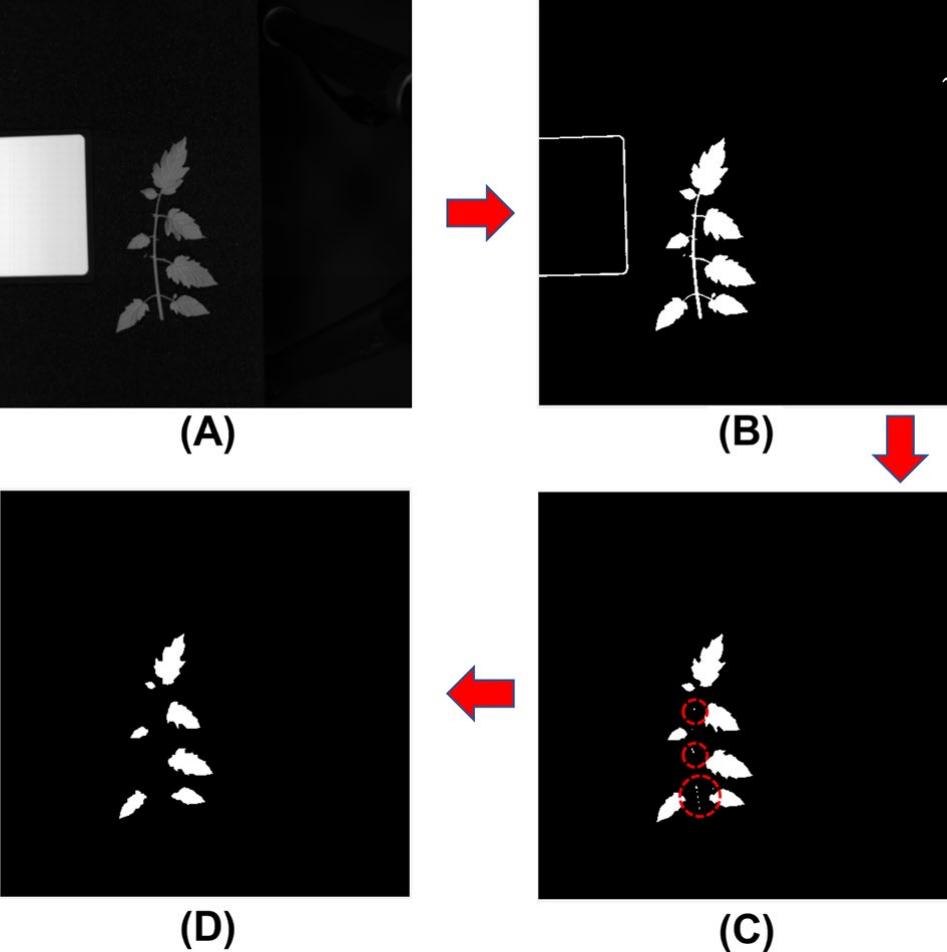
Spectral extraction process for hyperspectral images: (**A**) 780 nm grayscale image; (**B**) Threshold segmentation of background and leaf to obtain a binary image; (**C**) Removal of the border of the white reference panel and stem region by using image morphological analysis; (**D**) Removal of residual noise to obtain the mask image.

The obtained hyperspectral images were imported into MATLAB software to obtain grayscale maps with 204 bands. The clear images of the leaves were selected for constructing the mask images. The process of spectral extraction based on hyperspectral images was shown as Fig. 1: Step 1: according to the difference between the sample and background reflectance, a single-band image of the tomato leaf region with a high brightness was extracted. In this research, the grayscale map of the 780 nm band was selected for subsequent spectral data extraction (Fig. 1A); Step 2: according to the threshold range of reflectance in the sample area (50–200), the grayscale value of the map was set to 255, and the rest of the area was set as 0 to obtain a binary image (Fig. 1B); Step 3: erode image processing was performed on the binary image to remove the stem area and the border of the white reference panel (Fig. 1C); Step 4: the connection were marked and the areas with less than 100 pixel points were removed to eliminate the noise while retaining the leaf information (Fig. 1D). The final image obtained was the mask image.

**Figure 2:**
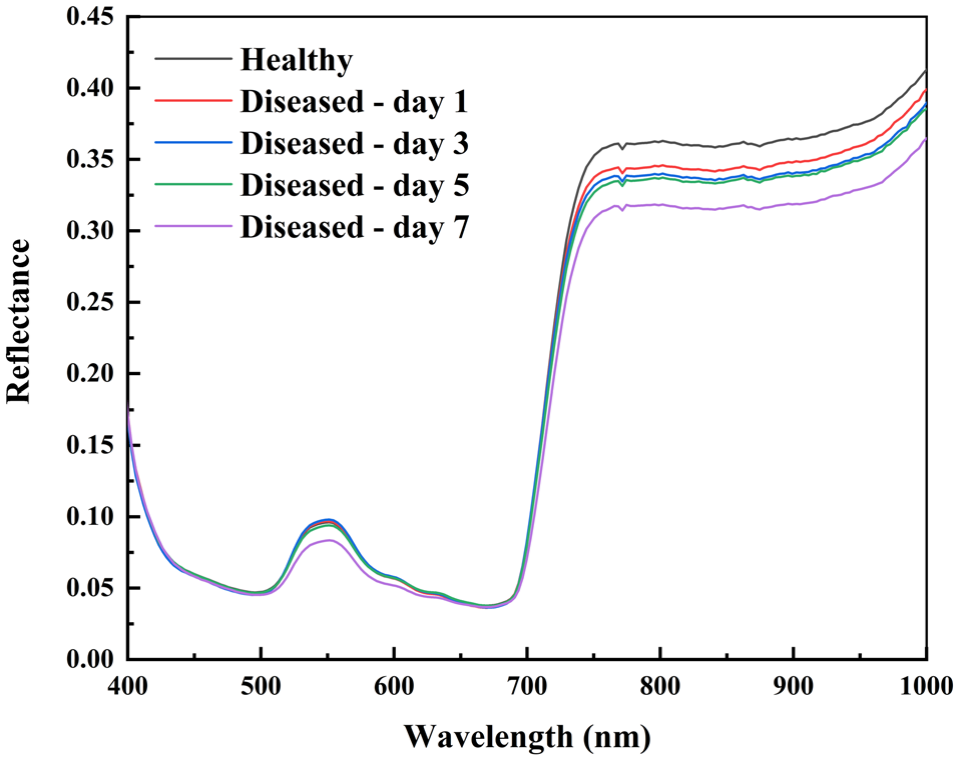
Average spectral reflectance curves of healthy and infected samples.

Based on the sample area determined from the mask image, the continuous spectrum under each pixel point in the sample area within the original hyperspectral map was extracted, and the average spectrum of all pixel points in each leaf was calculated. Figure 2 plots the average spectral reflectance curves of healthy and infected samples at different stages, showing that the spectral curves have similar trends. There is a clear reflectance peak near 555 nm, which is the strong reflectance peak of chlorophyll; a clear trough near 680 nm, which is caused by the strong absorption of chlorophyll^21^; and 500–600 nm reflects the greenness of the leaves. There is an exponential increase in reflectance from 680 to 750 nm, which is a plant-specific red-edge phenomenon. In the near-infrared band (750–1000 nm), the reflectance of healthy samples is higher than that of infected samples, and the reflectance of infected samples decreases with infection duration, which is caused by the destruction of plant cell tissues.

### Spectral analysis

**Figure 3:**
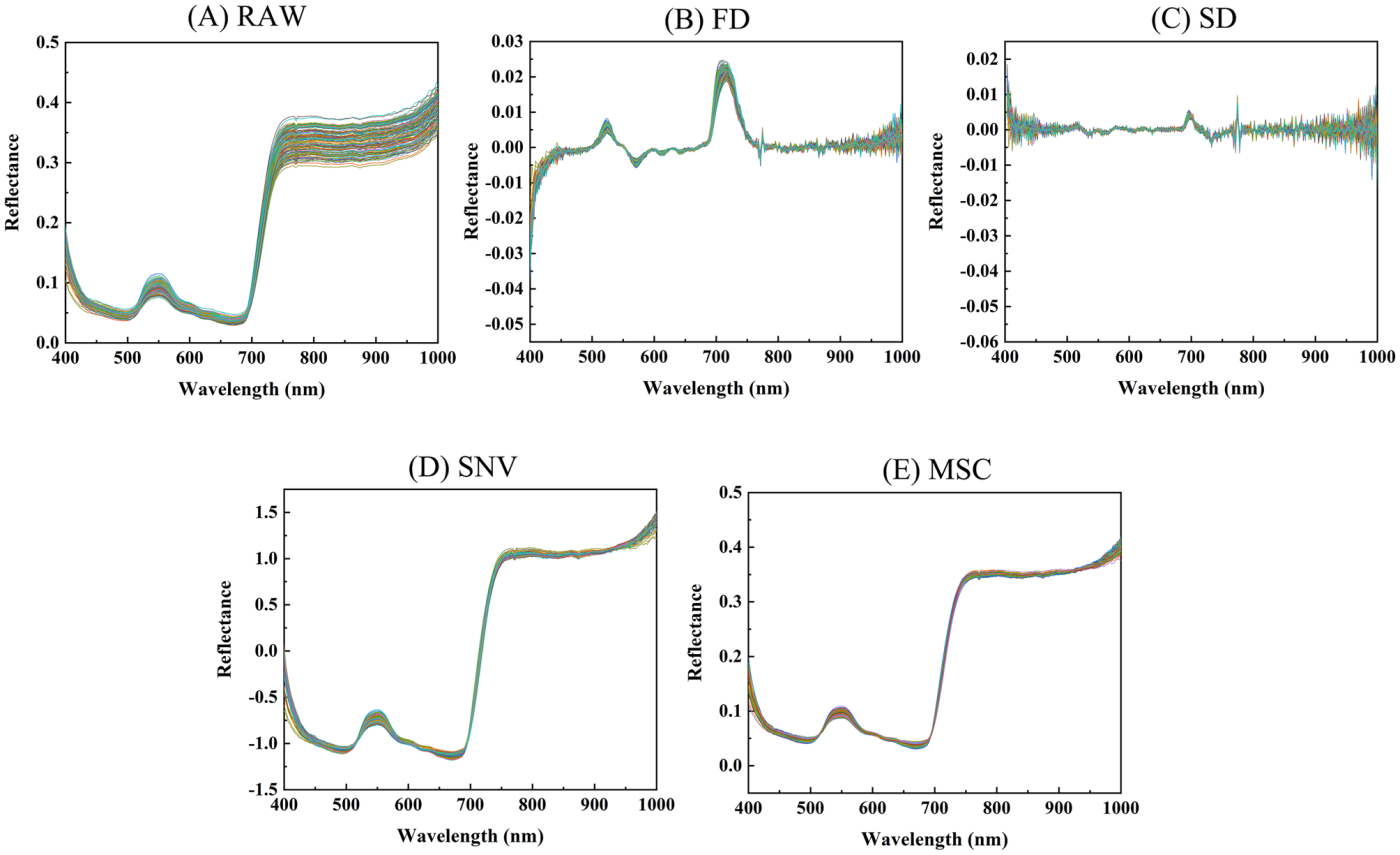
Spectra of tomato leaves: (**A**) Raw spectra; (**B**) FD: raw spectra after first derivative; (**C**) SD: raw spectra after second derivative; (**D**) SNV: raw spectra after standard normal variate; (**E**) MSC: raw spectra after multiplicative scatter correction.

The original spectra were preprocessed by using first derivative (FD), second derivative (SD), standard normal variant (SNV), and multiplicative scatter correction (MSC), and the original and preprocessed spectral profiles are shown in Fig. 3. Comparing the original and preprocessed spectral curves shows that the noise and redundant information were eliminated to some extent in the preprocessed spectral curves. Among them, the treated of FD and SD spectral curves reflected the rate of change of the original spectra and showed more in-depth information, but there is some noise in the 400–500 and 900–1000 nm bands. The SNV-processed and MSC-processed spectral curves possessed smoother spectral curves and eliminated the effects of baseline translation and nonlinear shift, retaining the original spectral trends. Although the SNV-processed and MSC-processed spectral curves made the spectral information tighter, there is still a certain amount of noise near the 1000 nm band.

### Classification results of multiple days

#### Classification results based on full wavelengths

**Figure 4:**
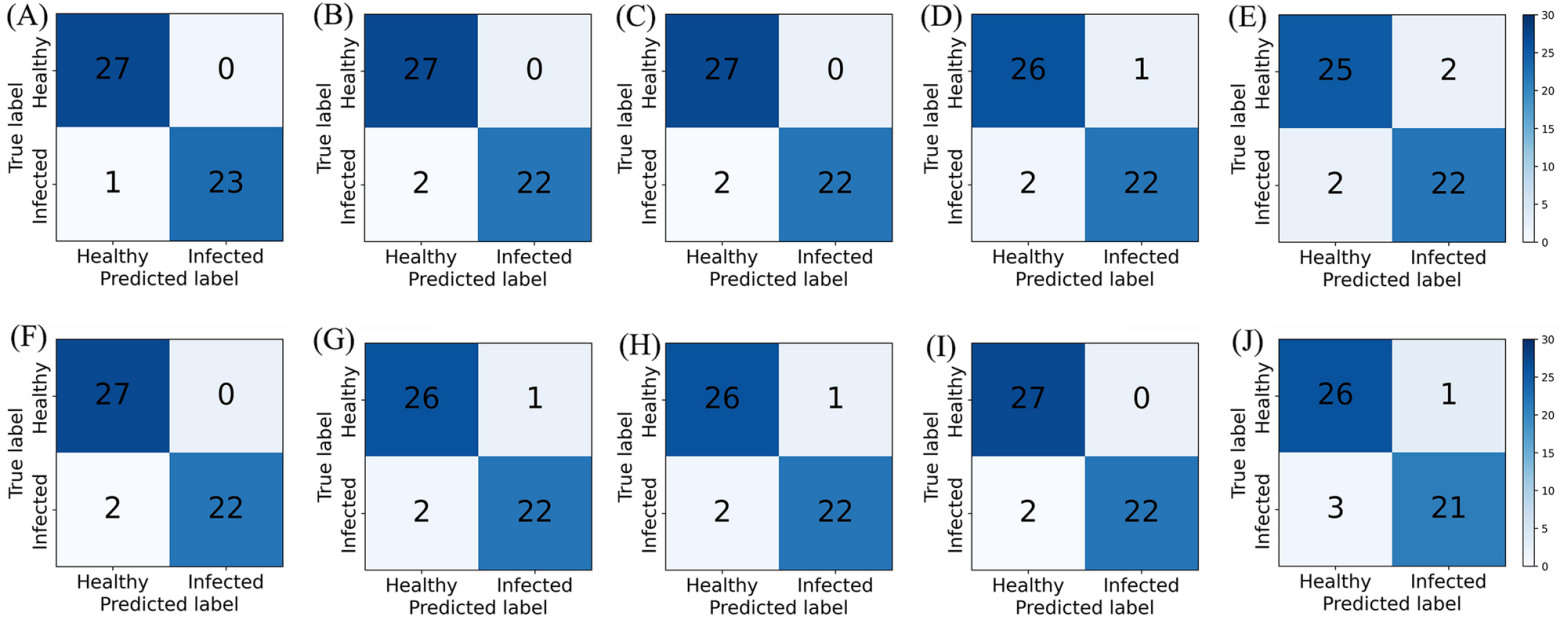
Confusion matrices of the PLS-DA and SVM models with different preprocessing methods. (**A**) RAW-PLS-DA. (**B**) FD-PLS-DA. (**C**) SD-PLS-DA. (**D**) SNV-PLS-DA. (**E**) MSC-PLS-DA. (**F**) RAW-SVM. (**G**) FD-SVM. (**H**) SD-SVM. (**I**) SNV-SVM. (**J**) MSC-SVM.

Due to the complexity of the natural growth environment, the severities of diseased tomato leaves were often different even in the same environment. Hence, these experiments were conducted to summarize the spectral data of healthy and infected samples under different incubation durations and to construct the detection model of *S. sclerotiorum* infection on tomato. Kennard-Stone (KS) algorithm^22^, which is the most widely used technique for training set design, resulted in models with best prediction performance with unknown data. Via using the KS algorithm, the samples were divided into the training sets and the test sets in a ratio of 4:1. The spectra were combined with different preprocessing methods for modeling and analysis to select the best spectral preprocessing method. Table 1 shows the classification results of the PLS-DA and support vector machine (SVM) models under different preprocessing methods. The accuracy of the calibration set of the SVM model was higher than that of the PLS-DA model, which could reach 100%. But the prediction performances were just a little bit different between the prediction sets of the two models, with the accuracy, sensitivity, and specificity reaching over 87.5%. Figure 4 shows the confusion matrices of different preprocessing methods, where A ~ E are the classification results of the PLS-DA model, and F ~ J are the classification results of the SVM model. Overall, the PLS-DA and SVM models have a higher probability of distinguishing infection as healthy than healthy as infection. By looking at the corresponding misjudgment results, it is found that the two infection spectra are the spectra of the first day of infection, which are like the healthy spectra. Compared to results with other pretreatment methods, accuracy with MSC pretreatment were poor.

**Table 1:** Classification results of the PLS-DA and SVM models with different preprocessing methods.

Type	Pre	Calibration set	Prediction set		
		Accuracy (%)	Accuracy (%)	Sensitivity (%)	Specificity (%)
PLS-DA	RAW	0.9877	0.9804	0.9583	1
	FD	0.9877	0.9608	0.9167	1
	SD	0.9877	0.9608	0.9167	1
	SNV	0.9877	0.9412	0.9167	0.9630
	MSC	0.9877	0.9216	0.9167	0.9259
SVM	RAW	1	0.9608	0.9167	1
	FD	1	0.9412	0.9167	0.9630
	SD	1	0.9412	0.9167	0.9630
	SNV	1	0.9608	0.9167	1
	MSC	1	0.9216	0.8750	0.9630

To better validate the prediction performance of the model, three images were extracted from both the healthy and diseased leaf images and the spectral information of all the pixel points within the leaf regions were extracted as the validation set of the model. The prediction results of the validation set are shown in Table 2. For both the PLS-DA and SVM models, the prediction accuracy on the validation set decreased. This is due to the model construction process using the average spectral data of the leaf as the calibration set, while the full spectrum of the leaf was used for the validation set, which indicates that there are large spectral differences between different parts of the leaf. Comparing the different preprocessing models, the SD preprocessed model showed the worst results, the SNV preprocessed model was stable with an average accuracy of up to 89.89%, while and the average accuracy of the SNV-SVM model reached 91.60%.

**Table 2:** Discriminant results of the PLS-DA and SVM models based on the full spectrum (Accuracy (%)).

Type	PLS-DA					SVM				
	RAW	FD	SD	SNV	MSC	RAW	FD	SD	SNV	MSC
Healthy A	0.7397	0.8531	0.7684	0.8451	0.7526	0.8164	0.7586	0.6424	0.8705	0.8059
Healthy B	0.7268	0.8246	0.6765	0.9089	0.6191	0.8909	0.6554	0.6069	0.9272	0.9460
Healthy C	0.4353	0.8521	0.7093	0.8570	0.7122	0.7403	0.7434	0.6505	0.8847	0.8905
Infected D	0.8214	0.7117	0.6522	0.9506	0.8338	0.8659	0.6662	0.5866	0.9566	0.9118
Infected E	0.7539	0.8031	0.6606	0.9117	0.8601	0.9225	0.8233	0.7150	0.9330	0.9076
Infected F	0.6349	0.7722	0.7170	0.9202	0.8431	0.7554	0.6878	0.6206	0.9243	0.9199
Average accuracy (%)	0.6853	0.8028	0.6973	0.8989	0.7702	0.8319	0.7224	0.6370	0.9160	0.8970

#### Classification results based on feature wavelengths

**Figure 5:**
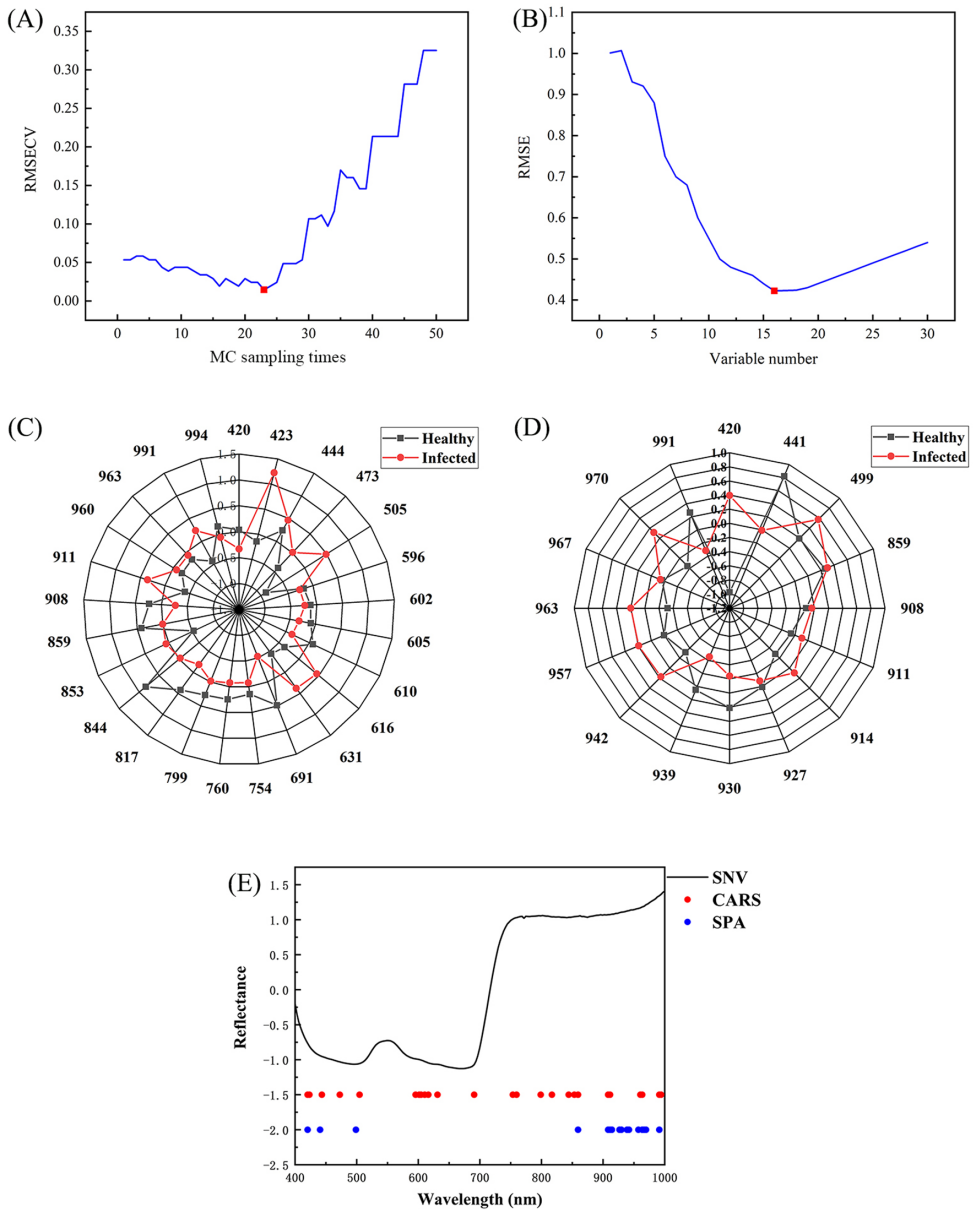
(**A**) MC sampling times versus RMSECV of CARS model. (**B**) Variable number versus RMSE of SPA models. (**C**) Plot of the band scores of healthy vs. infected samples after CARS band screening of the PLS-DA model. (**D**) Plot of the band scores of healthy vs. infected samples after SPA band screening of the PLS-DA model. (**E**) SNV preprocessing-based screening of CARS and SPA bands.

Band screening can reduce the influence of redundant and noisy information in the spectra, benefiting to improve the speed and accuracy of the model. The CARS and SPA were used for band screening of the best preprocessed spectra. Figure 5A shows the change of the RMSECV with the increase of MC sampling times in the CARS algorithm. In the initial stage, the RMSECV gradually decreased with the increase of MC sampling times due to the elimination of many variables irrelevant to prediction. With the further increase of sampling times, RMSECV increased, indicating that some important variables in the spectrum were eliminated. Therefore, the best feature variables were obtained under the 23rd MC sampling. Figure 5C indicates the key band scores based on the healthy and infected PLS-DA model samples screened by CARS, in which the latitude and longitude are the score value and wavelength, respectively. Evidently, there were differences between the healthy and infected samples at the 423, 505, 616, 631, 691, 844, and 911 nm bands. Meanwhile, Fig. 5B shows the variable number versus RMSE of SPA models. At the beginning, the RMSE decreased with the increase of the relevant variables. When the number of variables was 16, the RMSE reached the lowest value, and the optimal number of characteristic variables was 16. Figure 5D shows the differences between the healthy and infected samples after SPA screening of the 420, 441, 939, 963, 970, and 991 nm bands. CARS and SPA detected the overlapping bands at 420, 859, 908, 911, and 963 nm and decrease the number of wavelengths to 25 and 16 bands, respectively. The selected bands are shown in Fig. 5E.

**Figure 6:**
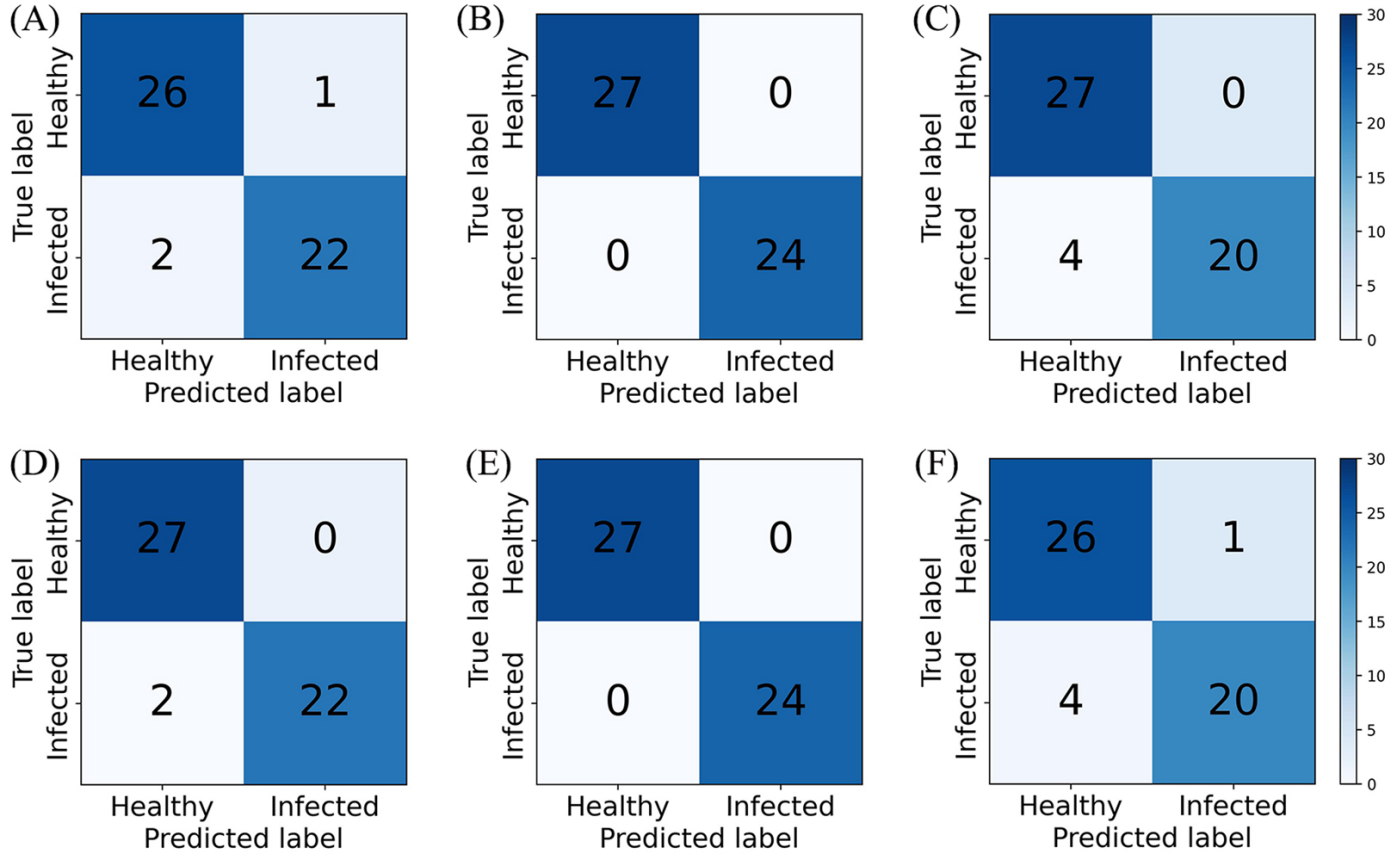
Confusion matrices of the PLS-DA and SVM models based on band screening under SNV preprocessing. (**A**) SNV-PLS-DA. (**B**) SNV-CARS-PLS-DA. (**C**) SNV-SPA- PLS-DA. (**D**) SNV-SVM. (**E**) SNV-CARS-SVM. (**F**) SNV-SPA-SVM.

To further verify the validity of the band screening, PLS-DA and SVM models were developed by using the key band screening base on CARS and SPA, separately, and were compared with the corresponding best preprocessed full-spectrum models. The comparison results are shown in Table 3. Compared with the full-spectrum model, the prediction accuracy, sensitivity, and specificity of the PLS-DA model based on the CARS band screening were improved to 100%, while the performance of the SVM model based on the CARS band screening was 100% in all cases. However, the performance of both models after the SPA band screening decreased. Figure 6 shows the confusion matrices of the PLS-DA and SVM models based on band screening under SNV preprocessing. As shown, Fig. 5A and D are the results of the SNV model, while Fig. 5B and E are the results of the CARS model. Figure 5C and F show the results of the SPA model. Comparing to the results of the SNV model, the CARS model could accurately predict healthy samples and infected samples, while the predictions of false-healthy were happened in SPA model. In this sense, the selections of CARS are more reasonable.

**Table 3:** Classification results of the PLS-DA and SVM models based on band screening under SNV preprocessing.

Pre	Model	Calibration set	Prediction set		
		Accuracy (%)	Accuracy (%)	Sensitivity (%)	Specificity (%)
SNV	PLS-DA	0.9877	0.9412	0.9167	0.9630
	SVM	1	0.9608	0.9167	1
SNV-CARS	PLS-DA	0.9873	1	1	1
	SVM	1	1	1	1
SNV-SPA	PLS-DA	0.9552	0.9216	0.8333	1
	SVM	0.9484	0.9020	0.8333	0.9630

To better evaluate the model performance, the validation set was predicted by using the above models. The results of model validation and operation speed are shown in Table 4. The model validation accuracy after band screening, especially for the SNV-SPA-PLS-DA model, experienced a reduction of 20%. A comparison between CARS and SPA highlighted that the model validation based on CARS was better, with the average accuracy of 87% and the speed increasing by an average of 3.5%. When the CARS-PLS and CARS-SVM models were compared, the accuracy of both was 87%, but CARS-SVM model ran 1.1% faster.

**Table 4:** Discrimination results of the PLS-DA and SVM models based on SNV preprocessing under band screening and full spectrum (Accuracy (%)).

Type	SNV-PLS-DA			SNV-SVM		
	Full spectra	CARS	SPA	Full spectra	CARS	SPA
Healthy A	0.8451	0.9233	0.7239	0.8705	0.9210	0.8846
Healthy B	0.9089	0.8570	0.6402	0.9272	0.8544	0.8894
Healthy C	0.8570	0.8953	0.7389	0.8847	0.8944	0.9293
Infected D	0.9506	0.9138	0.6870	0.9566	0.9158	0.8418
Infected E	0.9117	0.8817	0.6798	0.9330	0.8855	0.8570
Infected F	0.9202	0.8022	0.6841	0.9243	0.8058	0.8010
Average accuracy (%)	0.8989	0.8788	0.6923	0.9160	0.8795	0.8671
Average run time (s)	1.98	1.80	1.80	1.82	1.78	1.91

### Visualization of classification results

**Figure 7:**
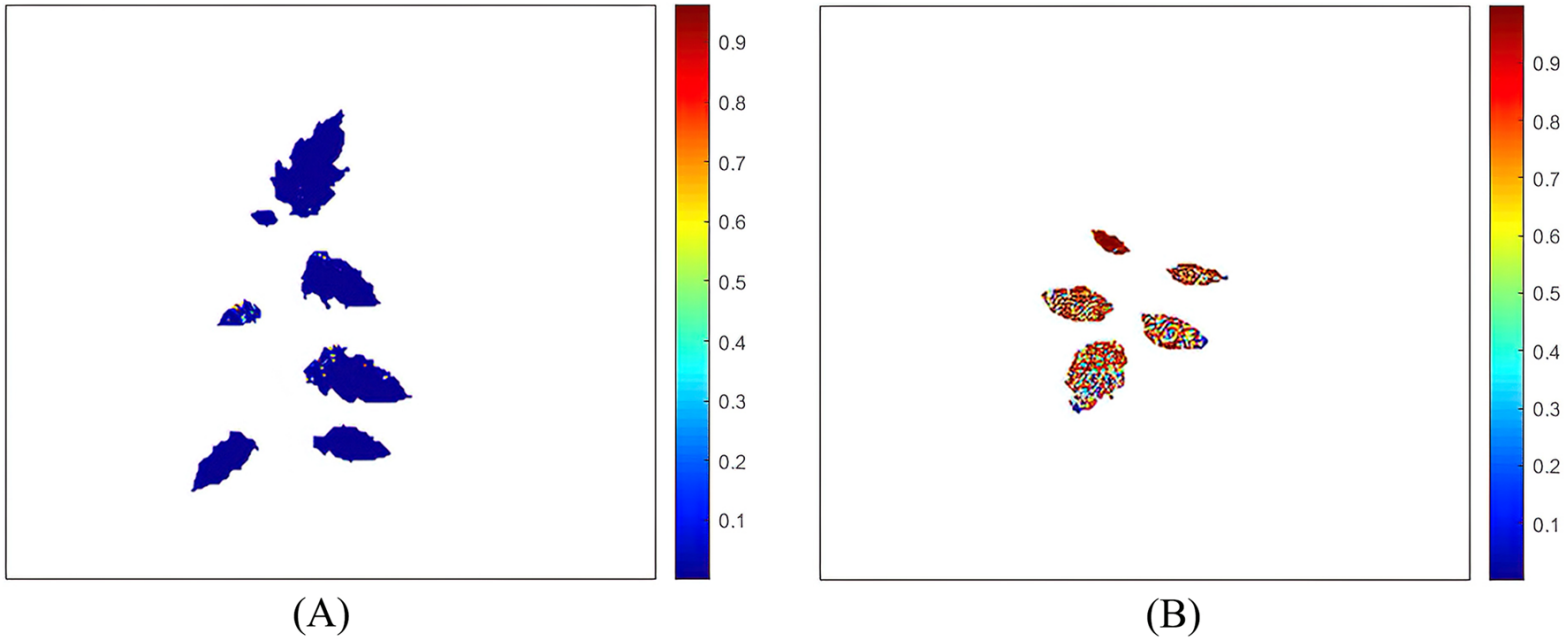
Visualization of leaf infection: (**A**) Healthy A; (**B**) Infected D (corresponds to healthy A and infected D in Table 4).

Due to the SNV-CARS-SVM model possessing a good performance stability, fast running speed, and high accuracy, the results of this model were used for visualization to observe tomato *S. sclerotiorum* infection more intuitively. This model is a binary discrimination model and cannot visualize the degree of leaf infection. Therefore, the probability function of the SVM model was introduced to visualize the probability of leaf infection. The visualization result is shown in Fig. 7. Colors closer to red indicate higher probability of infection, and colors closer to blue indicate higher probability of being healthy.

**Figure 8:**
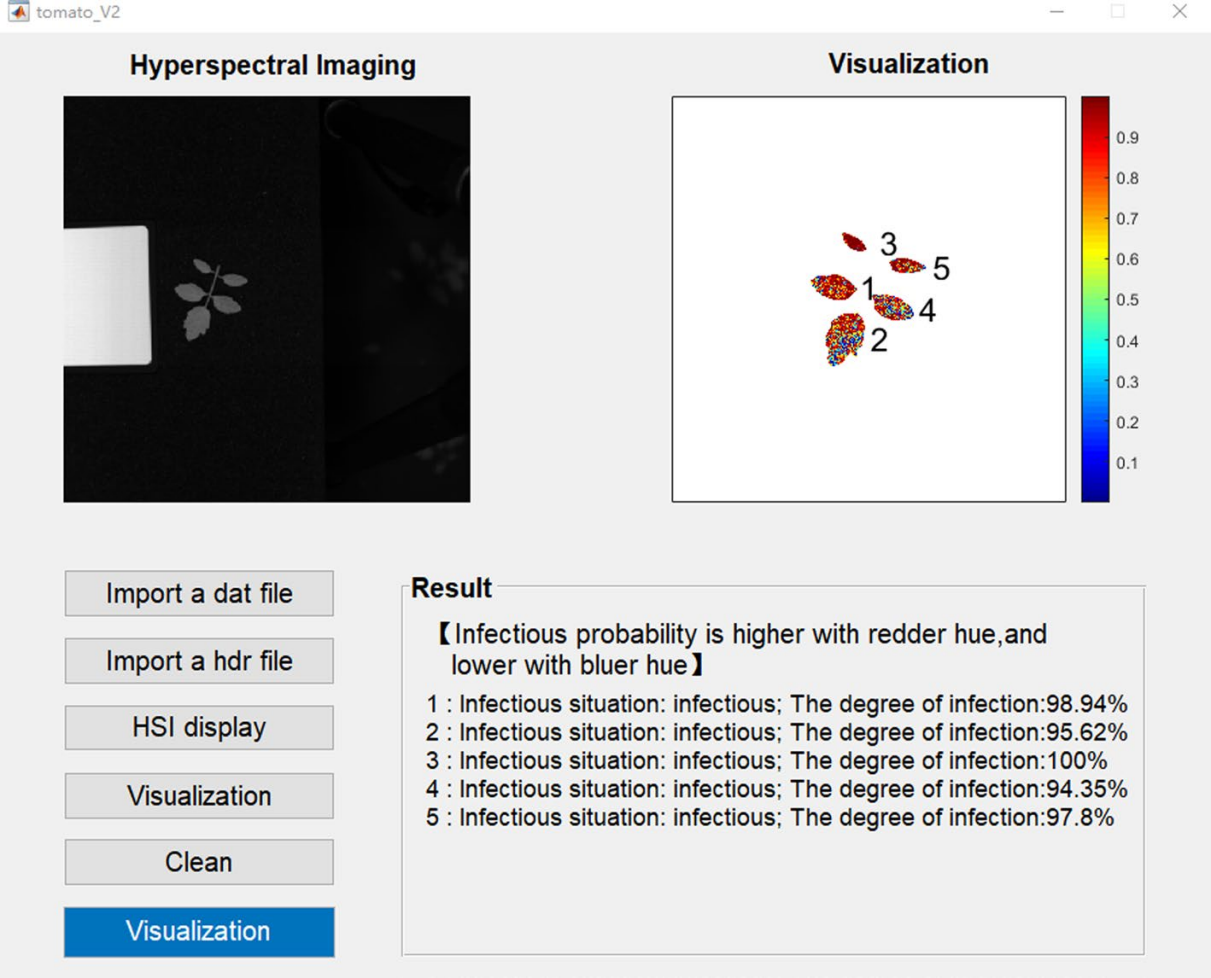
Visualization software interface.

To achieve online monitoring for the timely observation of the infection trends of leaves, the early warning and diagnostic visualization software for tomato *S. sclerotiorum* based on hyperspectral imaging was developed. As shown in Fig. 8, the software consisted of three parts: importing hyperspectral image files, displaying results, and clearing records. Hyperspectral image processing, spectral extraction, model analysis, and visual display were integrated and developed into a graphical user interface. The visual discriminatory results can be derived by clicking the corresponding buttons according to the text, and the discriminatory information of each leaf is displayed in the result box. According to the results of the SNV-CARS-SVM model in the validation set, which the lowest accuracy rate is 80.58%, there will be a maximum error of 20%. When the infected area of the leaf reaches 20%, infection is displayed and the corresponding infected area is shown. The opposite is shown as healthy.

## Conclusion

In this study, based on the hyperspectral imaging of tomato leaves infected with *S. sclerotiorum,* the feasibility of the hyperspectral imaging-based detection of tomato white mold. Since different levels of diseased tomato leaves are usually present in the same environment, the data from healthy and infected samples with different incubation periods were mixed. Four different spectral preprocessing methods (FD, SD, SNV, and MSC) were used to select the optimal preprocessing method for *S. sclerotiorum* detection by comparing the results of the calibration, prediction, and validation sets. Then, the CARS and SPA band screening methods were used to obtain the key waveform information to re-build the model. Finally, the model with a high stability, good performance, and fast computing speed was selected. To observe the leaf infection more intuitively, the leaf pseudo-color map was established. Simultaneously, the image processing, spectral extraction, model analysis, and visualization analysis were integrated for the early warning and diagnostic visualization of tomato leaf *S. sclerotiorum* disease. Based on the results of the study, the conclusions can be drawn as follows: (1) Hyperspectral imaging technology is feasible for the early detection of tomato *S. sclerotiorum* infection. (2) Four different spectral preprocessing methods (FD, SD, SNV, and MSC) achieved an accuracy of over 92% for the prediction sets of the PLS-DA and SVM models. However, for the validation set, the performance stability of the SNV preprocessed model was the best, displaying that the average accuracy reached over 89% and the average accuracy of the SNV-SVM model reached 91.6%. (3) Based on the SNV preprocessing method, the accuracy of the validation set after CARS and SPA band screening decreased, but the model operation speed improved by 4%. Within this, the CARS model had a better effect, revealing the model accuracy maintained at 87% and operation speed improved by 3.5%. (4) The hyperspectral imaging-based visualization software for early warning and diagnosis of tomato *S. sclerotiorum* contributes to visually observe the infection trend of the leaves. This can facilitate the targeted diagnosis and the treatment directions for the plant and provides some research ideas for the online in situ detection of tomato *S. sclerotiorum* infection.

## Materials and methods

### Hyperspectral imaging system

**Figure 9:**
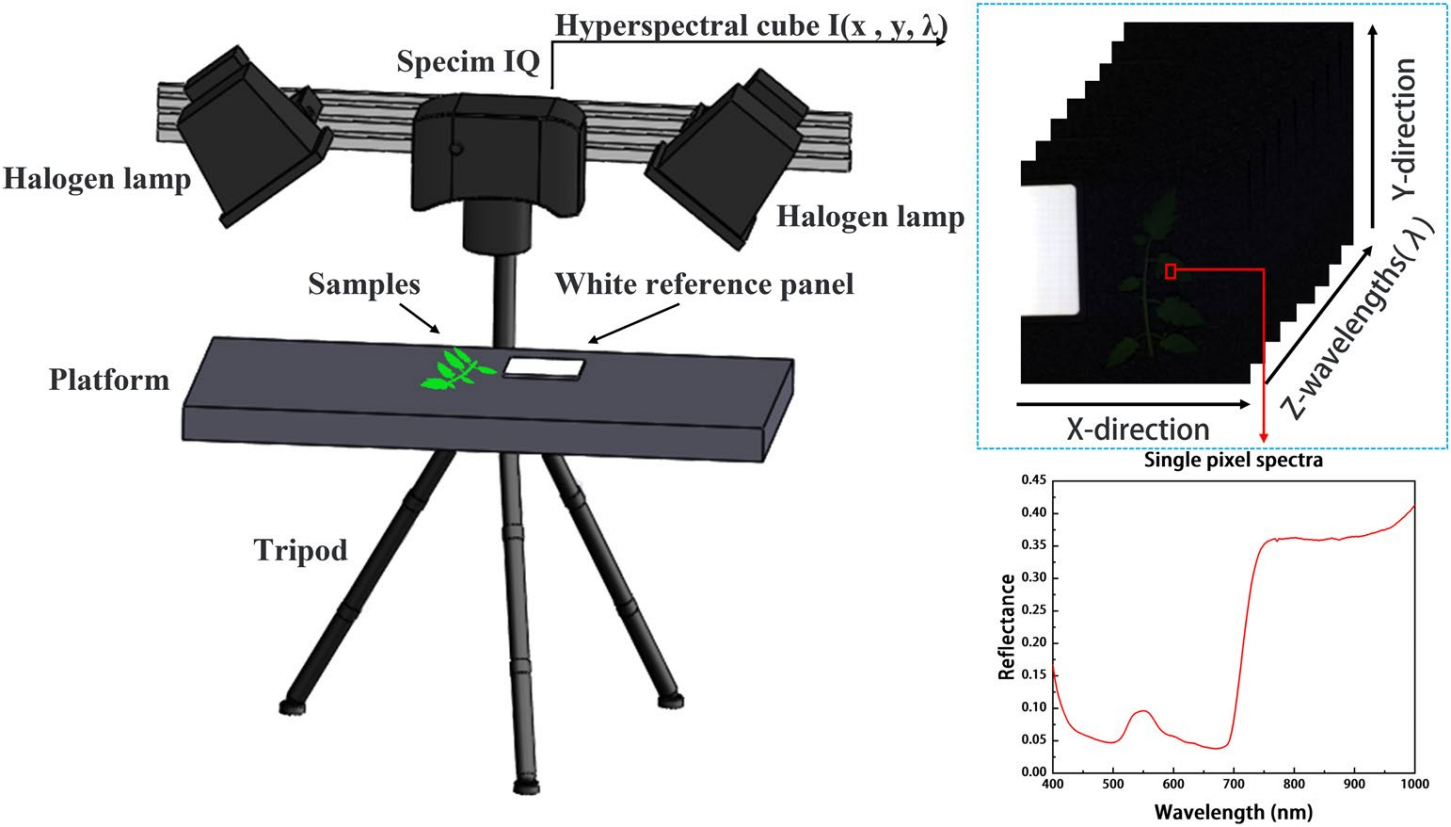
3D structure of the hyperspectral imaging system.

The hyperspectral image acquisition equipment used in this study was the SPECIM IQ portable hyperspectral imaging system (Spectral Imaging Ltd, Finland), which consists of a hyperspectral camera, halogen light source, tripod, and white reference panel (Fig. 9). The hyperspectral camera was a SPECIM IQ handheld intelligent camera with two 150 W halogen lamps to simulate sunlight in a natural environment. During image acquisition, black-white calibration was automatically completed. The image resolution of the camera was 512 × 512. The spectral range of the acquired image was 400–1000 nm and spectral resolution was 3 nm. From this, 204 spectral bands can be obtained, which is sufficient for effective spectral data analysis. In this study, MATLAB R2018b software was used for image processing, spectral extraction, data analysis, and software development.

### Sample preparation

Tomato samples were cultured at Guangdong Institute of Modern Agricultural Equipment. For the experiment, tomato plants with roughly the same growth status and a good leaf flattening status were selected. The leaves were artificially inoculated with *S. sclerotiorum* spore suspension, for which the spore suspension was sprayed on the leaves at a close range to ensure that the infected area completely covered the leaves. After spraying, the plants were placed in an airtight environment with humidity of 95% and temperature of 25 °C for 24 h to ensure that maximum infection could occur^23^. Following this, inoculated and uninoculated samples were placed in different areas and irrigated with an appropriate volume of water daily. Hyperspectral images of infected and healthy leaves were collected on days 1, 3, 5, and 7 after inoculation. A total of 139 infected (34, 32, 35 and 38 leaves on days 1, 3, 5 and 7, respectively) and 145 healthy leaves (34, 40, 33 and 37 leaves on days 1, 3, 5 and 7, respectively) were collected.

### Hyperspectral image and spectral acquisition

To prevent interference from extraneous light sources other than the halogen lamps, image acquisition was performed in the dark. During image acquisition, the leaf was cut and placed flat at a vertical distance of 30 cm below the hyperspectral camera. The imaging background was black, and a 99% reflectance white reference panel was placed next to the leaf for the hyperspectral camera to perform automatic image correction. The camera exposure time was set to 20 ms.

The acquired hyperspectral image was imported into MATLAB software, and a single-band image of the tomato leaf region with a high brightness was extracted according to the difference between the sample and background reflectance. Then, the mask image was built based on binarization, and the region of interest in the mask image was selected to obtain the hyperspectral data of the sample. In this study, a complete leaf was selected as the region of interest, from which the average spectrum of each pixel point in the region was taken as the spectral data of the leaf.

### Spectral preprocessing

To reduce the anomalies in the spectral information caused by interfering factors, such as the plant's growth status, a Monte Carlo-partial least squares (MC-PLS)^24^ method was used to reject the outlier samples by considering the sample and spectral information. This method can reduce the risk caused by masking effects, while improving the accuracy and stability of the model^25^. In the MC-PLS process, the samples were randomly divided 500 times and the PLS model was constructed separately. The mean and standard deviation of each sample were calculated based on the predicted residuals of each group of samples. Outlier samples with a mean and Std exceeding their median values by 3 times were eliminated to obtain 127 infected leaves (32, 30, 30 and 35 leaves on days 1, 3, 5 and 7, respectively) and 132 healthy leaves (32, 38, 30 and 32 leaves on days 1, 3, 5 and 7, respectively).

To reduce the effects of the instrument noise, measurement environment, and sample conditions on the raw spectra, the raw spectra underwent FD, SD, SNV and MSC processing after the outliers were removed. This made the spectral information with a high correlation more prominent, thus improving the accuracy of the subsequent modeling analysis.

### Characteristic wavelength selection

The dataset collected from the hyperspectral images consisted of continuous multi-wavelength spectral data, which often contain some redundant information due to collinearity between the data. If the modeling analysis was performed on the entire band, this would not only affect the efficiency of the modeling, but also affect the model’s detection performance. Therefore, before modeling and analyzing the spectral data, characteristic wavelengths need to be extracted to reduce the redundant information and obtain a simpler more efficient detection model.

#### Successive projections algorithm (SPA)

SPA is a characteristic wavelengths selection method that can filter important information from the complicated spectral data and eliminate the collinearity between the different wavelengths^26^. First, SPA randomly selects the initial iteration vector, compares the projection vector size with the unselected wavelength variables, selects the largest projection vector as the characteristic wavelength for the initial value of the next iteration, and then the cycle is repeated. To obtain a set of wavelengths for selection, the cycle ceases when the number of iterations is greater than the number of wavelengths. These wavelengths were used to build a correction model. Under the prerequisite of not losing prediction accuracy, the final characteristic wavelength was selected according to the minimum root mean square error (RMSE)^27^.

#### Competitive adaptive reweighted sampling (CARS)

CARS is an algorithm that performs wavelength screening based on regression coefficients to obtain high-quality characteristics to build stable and accurate calibration models^28^. It is used to build a PLS model via Monte Carlo sampling by calculating the absolute weights of the regression coefficients of the wavelength variables at each Monte Carlo sampling point. Furthermore, after removing wavelength variables with low absolute values of regression coefficients according to the exponential decay function (EDF), CARS performs adaptive reweighted sampling (ARS) to screen out significant wavelengths. The above steps were repeated to obtain N wavelength subsets, then a PLS model was constructed, and finally cross-validation was used to select the subset with the lowest root mean square error of cross validation **(**RMSECV) as the best wavelength combination^29^.

### Model construction and validation

#### Classification methods

In this study, PLS-DA and SVM were used as classification methods. PLS-DA is a linear discriminant algorithm that obtains the classification information of the samples via the partial least squares (PLS) algorithm. This method constructs a regression model between the independent variables and classification information to obtain the characteristic variables that are highly correlated with the classification information and achieve sample classification^30^. SVM is an algorithm for the binary classification of samples based on supervised learning. The main idea is to find the separation hyperplane that can correctly classify the sample data with the largest geometric interval. Sample data that are difficult to classify can be mapped to the high-dimensional space to achieve sample classification. For data operations in high-dimensional space, the introduction of a kernel function is required, of which the most used is the radial basis function (RBF). To improve the model performance, grid search^31^, genetic algorithm^32^, particle swarm optimization^33^, and other parameter optimization algorithms were used in the modeling process to identify the optimal solution for the penalty coefficient c of the SVM and regularization coefficient g of the RBF.

#### Validation of prediction models

Model evaluation is used to measure the effectiveness of different models in parameter space and feature extraction. Classification model performance is generally evaluated for accuracy, sensitivity, and specificity of the prediction set^34^. Accuracy is the ratio between the number of correctly identified samples and the total number of samples. As two important indicators in clinical diagnosis, Sensitivity and specificity are the ratio of positive and negative samples that are correctly classified, respectively. The closer the accuracy, sensitivity, and specificity are to 1, the better the classification performance of the model.

1 $$Accuracy= \frac{TP+TN}{TP+FP+TN+FN}$$

2 $$Sensitivity= \frac{TP}{TP+FN}$$

3 $$Specificity= \frac{FP}{FP+TN}$$

where, TP denotes the number of positive samples correctly classified by the model, FN denotes the number of positive samples incorrectly classified by the model, TN denotes the number of negative samples correctly classified by the model, and FP denotes the number of negative samples incorrectly classified by the model.

**Figure 10:**
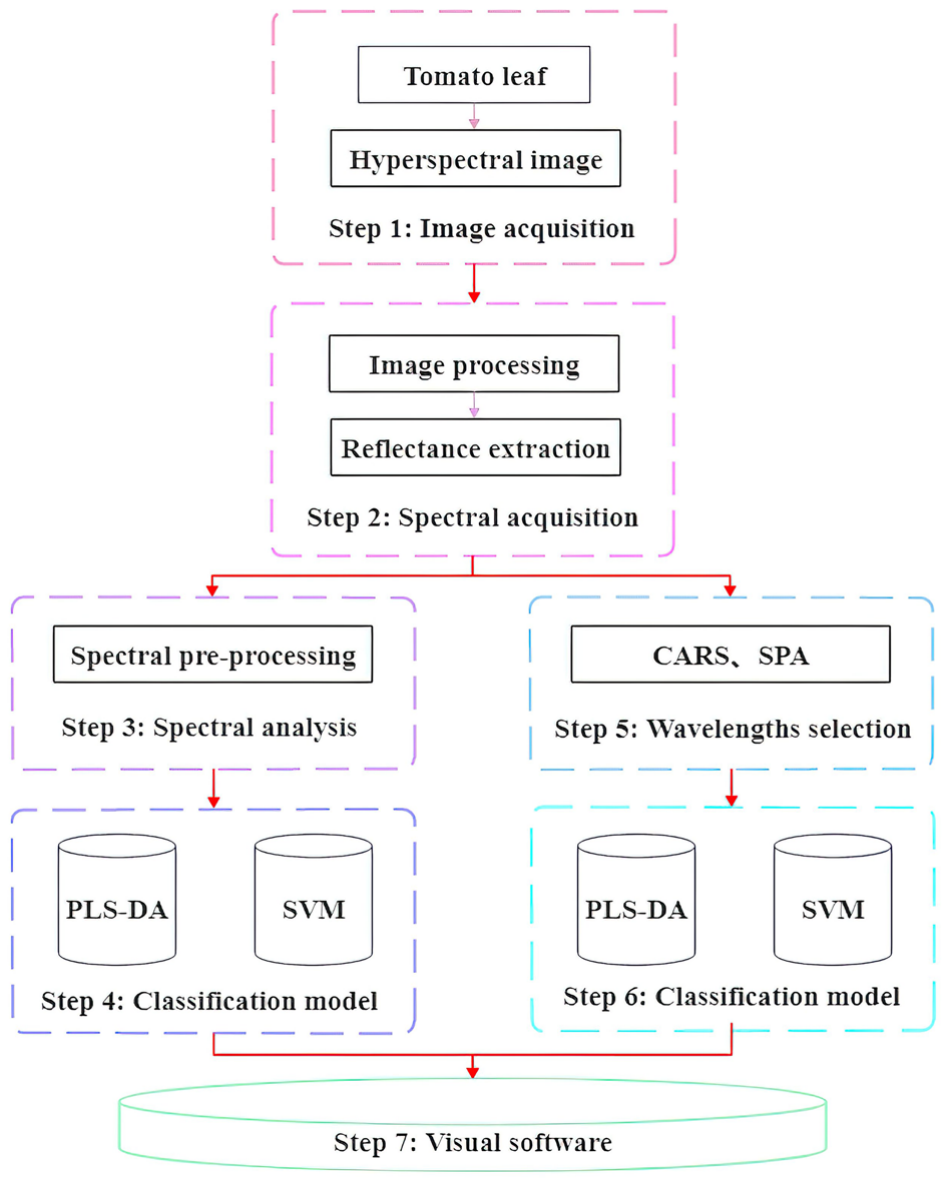
Flow chart of the main steps of hyperspectral imaging-based tomato leaf *Sclerotinia sclerotiorum* detection.

The computer specifications for the model construction and performance evaluation are as follows: operating system, Win10; processor, Intel (R) Core (TM) i5-8500; CPU, 3.00 GHz; RAW, 16G. The main steps of the study can be described in Fig. 10. After image acquisition and spectral extraction of tomato leaves, spectral preprocessing (FD, SD, SNV, MSC) were performed on the full spectrum. The PLS-DA model and SVM model were established based on spectral preprocessing, respectively. According to the best preprocessing method, the band screening (CARS and SPA) was performed based on the best preprocessing method. Then, the best model was derived from the modeling analysis based on the band screening. Finally, the visualization software was developed based on the best model.

## Data Availability

The data that support the findings of this study are available from the corresponding author upon reasonable request.
